# Guaiazulene derivative 1,2,3,4‐tetrahydroazuleno[1,2‐*b*] tropone reduces the production of ATP by inhibiting electron transfer complex II

**DOI:** 10.1002/2211-5463.13215

**Published:** 2021-09-21

**Authors:** Chieko Kasami, Jun‐ichi Yamaguchi, Hideki Inoue

**Affiliations:** ^1^ Department of Applied Bioscience Faculty of Applied Bioscience Kanagawa Institute of Technology Atsugi Japan; ^2^ Department of Applied Chemistry Faculty of Engineering Kanagawa Institute of Technology Atsugi Japan

**Keywords:** apoptosis, *C* *. elegans*, cancer, metabolism, mitochondria, OXPHOS

## Abstract

Molecularly targeted therapy has been used for treatment of various types of cancer. However, cancer cells often acquire resistance to molecularly targeted drugs that inhibit specific molecular abnormalities, such as constitutive activation of kinases. Even in cancer cells that have acquired resistance, enhanced anabolism, including the synthesis of nucleotides, amino acids and lipids, is common to normal cancer cells. Therefore, there is a renewed interest in effectively eliminating cancer cells by specifically targeting their abnormal energy metabolism. Multiple strategies are currently being developed for mitochondrial‐targeted cancer therapy, with agents targeting oxidative phosphorylation, glycolysis, the tricarboxylic acid cycle and apoptosis. In this study, we found that one of the guaiazulene derivatives, namely, 1,2,3,4‐tetrahydroazuleno[1,2‐*b*] tropone (TAT), inhibited the proliferation of cancer cell lines stronger than that of normal cells. In addition, we showed that TAT inhibited energy production in cancer cell lines, resulting in apoptosis. Analyses done in cancer cell lines and in the animal model *Caenorhabditis elegans* suggested that TAT acts on the mitochondrial electron transfer complex II and suppresses cellular energy production by inhibiting oxidative phosphorylation across species. These results suggest that TAT could represent a novel anticancer agent that selectively targets mitochondria.

## Graphical abstract

Here we demonstrate that a guaiazulene derivative, namely, 1,2,3,4‐tetrahydroazuleno[1,2‐*b*] tropone (TAT), inhibits the proliferation of cancer cell lines. TAT acts on mitochondrial electron transfer complex II by inhibiting oxidative phosphorylation, thereby suppressing cellular ATP production, which leads to cellular energy deprivation and cell death. These results suggest that TAT has the potential to be a novel anticancer agent that selectively targets mitochondria.

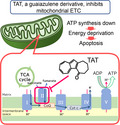

**Abbreviations**
FRAP: ferric reducing ability of plasma
MAPK: mitogen‐activated protein kinase
PARP: poly ADP‐ribose polymerase
ROS: reactive oxygen species
SDH: succinate dehydrogenase
TAT: 1,2,3,4‐tetrahydroazuleno[1,2‐b] tropone
UPR: unfolded protein response

Cancer can be described as a group of cells that have lost their ability to induce and control cell division because of genetic abnormalities. Loss of normal function as a result of genetic mutations is often followed by mutations in oncogenes, tumor suppressor genes and other genes, which, in turn, leads to cancer [1]. Both chemotherapy and molecularly targeted therapy are effective therapeutic methods. Chemotherapy focuses on cancer cell metabolic characteristics to inhibit or kill the cancer cells, but normal cells are also killed in this process [2]. Molecularly targeted cancer drugs are used to suppress the activity of cancer cells caused by specific genetic mutations, thereby lowering the likelihood of also killing normal cells. However, the signaling pathways involved in cancer are multifaceted. Thus, when the causative gene is unknown or the cancer is caused by multiple gene mutations, it is difficult to treat the cancer with molecularly targeted drugs [3].

The synthesis of biomolecules and the process of cell division cannot be sustained without a continuous supply of energy in the form of ATP. ATP is required for a variety of cellular activities, including transcription, translation and protein phosphorylation, which is necessary for signal transduction. Thus, actively dividing cancer cells require vast amounts of ATP, more than normal cells. Multiple hypotheses exist regarding the mechanism of energy metabolism in cancer cells; two are presented here. First, the classic Warburg effect describes the synthesis of ATP from the fermentation of glucose by anaerobic glycolysis. Warburg believed that accelerated anaerobic glucose metabolism through fermentation, rather than oxidative respiration, was the source of ATP for cancer growth [4]. It is widely accepted that glucose is the main carbon source for ATP synthesis in cancer cells. Second, cancer cells consume glucose generating ATP‐producing lactate (the Warburg effect), and neighboring cancer cells consume the secreted lactate to produce additional ATP under aerobic conditions via the tricarboxylic acid cycle and oxidative phosphorylation [5, 6, 7]. Therefore, targeting cancer‐specific abnormalities and/or unique mechanisms of metabolism may be an effective way to treat cancer.

Bonnet *et al*. [3] suggested that metabolic adaptations that occur in tumor cells can be used to selectively kill cancer cells by ‘normalizing’ the metabolic characteristics associated with cancer. Cancer cells are not homogeneous in nature, existing as a population of cells with different properties. Therefore, cancer cells that survive anticancer drug treatment targeting specific molecular aberrations develop resistance to these drugs. Anticancer drugs that target mitochondrial respiration, on which cells share a common energy dependence, may be effective in preventing the cells from developing resistance [7, 8, 9, 10, 11]. Metformin inhibits tumor growth by targeting mitochondria and blocking tumor‐specific metabolism by inhibiting oxidative phosphorylation, which the tumor is dependent on to make ATP. Metformin decreases mitochondrial respiration and increases the percentage of mitochondrial respiration devoted to deconjugation reactions. Metformin‐treated cells become energetically inefficient and show an increase in aerobic glycolysis and a decrease in glucose metabolism through the mitochondrial tricarboxylic acid cycle [12].

Plant natural compounds and their derivatives continue to serve as essential sources for drug development. In the oncology field, up to 80% of approved drugs are natural products or based on them [13, 14]. Chamomile has been one of the most commonly used herbal medicines since ancient times, and among its many benefits is its therapeutic effect on various types of inflammation, which is widely known empirically. The anti‐inflammatory effect is suggested to be due to the presence of azulene in the essential oil component of chamomile leachate [15, 16]. The emergence of azulene as a therapeutic agent was prompted by research on azulene derivatives, with guaiazulene and chamazulene being the two most effective. Guaiazulene and chamazulene have shown excellent therapeutic effects on asthma, rheumatism, x‐ray skin injuries, skin ulcers, chronic skin diseases, drug allergies and other inflammatory or allergic diseases [15, 17, 18]. These derivatives are also expected to be effective therapeutic agents [19, 20]. Sodium guaiazulene sulfonate is a water‐soluble form of guaiazulene, which has anti‐inflammatory, antiallergic, antiulcer and wound‐healing properties. Its mechanism of action is due to the inhibition of leukocyte migration and histamine release from mast cells without the pituitary–adrenal system, which is thought to be a direct local action on inflammatory tissue. Medicines based on sodium guaiazulene sulfonate have long been used as a treatment for oral inflammatory diseases and gastritis, and their efficacy and safety have been highly evaluated [21, 22, 23]. Among other azulene derivatives, 2‐acetylaminoazulene, diethyl 2‐chloroazulene‐1,3‐dicarboxylate and methyl 7‐isopropyl‐2‐methoxyazulene‐1‐carboxylate are relatively highly selective, and chlorination, more than fluoridation, of azulene has been found to increase tumor selectivity for cytotoxic activity [24, 25].

The bioactivity of the guaiazulene derivative 1,2,3,4‐tetrahydroazuleno[1,2‐*b*] tropone (TAT), an oxidized three‐membered azulene derivative, is not known [26]. Here, we examined the effect of TAT on cancer cell lines and the model animal *Caenorhabditis elegans* to determine whether TAT has selective anticancer potential and, if so, if it extends across species.

## Materials and methods

### Cell culture

HEK293 and HeLa cells were cultured in high‐glucose Dulbecco's modified Eagle's medium (Nacalai Tesque, Kyoto, Japan) supplemented with 10% FBS (HyClone, Cytiva Tokyo, Japan) and penicillin–streptomycin (Nacalai Tesque) in an incubator containing 5% CO_2_ at 37 °C. TIG‐1 fibroblasts, purchased from Japanese Collection of Research Bioresources (JCRB) Cell bank, were cultured in Eagle's minimal essential medium (EMEM) supplemented with 10% FBS and penicillin–streptomycin.

### *C. elegans* strains

All strains were maintained on nematode growth medium plates at 20 °C and fed with bacteria of the OP50 strain, as described by Brenner [27]. The alleles used in this study were N2 Bristol as the wild‐type and *feIS4* (*sur‐5p::luciferase‐gfp*), zcIS13 (*hsp‐6p::gfp*), *sek‐1(km4)* and *mev‐1(kn1)* as mutants. Worm strains were purchased from Caenorhabditis Genetics Center. The genetically modified strains used in this study do not confer any advantage on the animals or allow the animals to secrete or produce any infectious agents as a result of the genetic modification.

### Cell count

Cultured cells were seeded in a 96‐well plate at a density of 5000 cells per well. The following day, cells were treated with TAT (in DMSO) or DMSO and incubated for indicated times. Cell viability was monitored using Cell Counting Kit‐8 (WST‐8; Dojindo, Kumamoto, Japan) according to the manufacturer's instructions. Absorbance was measured at 450 nm. To investigate cell number, we assayed TAT or control‐treated cells in a 96‐well plate using Cell Normalization kit using Hoechst 33342 (Dojindo) according to the manufacturer's instructions. Cells were counted by fluorescence at 450 nm. The amount of intracellular ATP was measured by luminescent ATP assay solution (TOYO B‐net Inc., Tokyo, Japan). Detection of all of the earlier indicators was performed using Mithras LB940 plate reader (Berthold Inc., Calmbacher, German).

### Isolation of mitochondria and inhibition of ATP production

Mitochondrial extraction from cultured cells was performed according to the attached protocol using the mitochondria isolation kit (ab110170; Abcam, Cambridge, UK). *In vitro* ATP inhibition experiments were performed based on the method of Drew and Leeuwenburgh [28]. To test the amount of ATP, we added and incubated TAT and/or rotenone for 10 min at 37 °C to freshly isolated mitochondria in a reaction buffer. The inhibition in the rate of ATP production was determined by comparing the treated mitochondria with an equal portion of freshly isolated mitochondria from the same extract. The ATP level in the reaction mixture was measured with a luminometer after adding the luminescent ATP assay solution to the reaction mixture.

### Western blot

Animals were homogenized in sample buffer [65 mm Tris–HCl (at pH 6.8), 3% SDS, 10% glycerol and 5% 2‐mercaptoethanol] for immunoblotting. The primary antibody of this experiment includes PARP [poly ADP‐ribose polymerase, Cell Signaling Technology (CST), Danvers, MA, USA, #9542], Caspase‐9 (CST, #9504), Caspase‐7 (CST, #12827), β‐tubulin (ProteinTech, Rosemont, IL, USA, 10068‐1‐AP), GFP (green fluorescent protein, MBL Inc., Tokyo, Japan, M048‐3). The dilution factor for all antibodies is 1 : 1000.

### Lifespan analysis

Fourth larval stage animals or young adults were picked to nematode growth medium agar plates containing 5′‐fluoro‐2′‐deoxyuridine (TCI) to prevent the growth of progeny. Worms were tapped every 2 days and were scored as dead when they did not move after repeated taps with a pick. After scoring, they were transferred to new plates. Worms that crawled off the plate, exploded or died from internal hatching were excluded from analysis. *P* values were calculated by Student's *t*‐tests unless otherwise stated.

### Ferric reducing ability of plasma assay

The ferric reducing ability of plasma (FRAP) method was based on that of Benzie and Strain [29]. Standard solutions of iron (II) sulfate heptahydrate ranging in concentration from 0.25 to 8 mmol·L^−1^ were made. Solutions to make up the FRAP reagent were prepared: 300 mmol·L^−1^ acetate buffer, 10 mmol·L^−1^ TPTZ/HCl solution and 20 mmol·L^−1^ ferric chloride. A 96‐well plate was used for the assay; sap samples were diluted directly into wells. The FRAP reagent was produced using the acetate buffer, TPTZ–HCl and ferric chloride hexahydrate solution and then added to each well. Absorbance was measured directly at 620 nm.

### GC/MS sample preparation

The treated cells were washed twice with PBS, and then methanol was added to extract the suspension. Subsequently, 2‐isopropylmalic acid was added as a standard substance, followed by drying under reduced pressure. Samples were incubated at 37 °C with the addition of methoxyamine–pyridine (20 mg·mL^−1^) solution and *N*‐methyl‐*N*‐trimethylsilyltrifluoroacetamide (MSTFA) and converted to trimethylsilyl ether.

### GC/MS analysis

GC/MS was performed on an Agilent (Santa Clara, CA, USA) 6890N gas chromatograph coupled to an Agilent 5973 mass spectrometer. Each sample was introduced into an 8CB‐MS capillary column (Agilent), 30 m long × 0.25 mm inner diameter with 0.25‐μm film thickness. Automatic injections of 0.5‐μL samples were made, 1 : 30 splitting, into the GC inlet set to 230 °C. The thermal program began at 80 °C for 2 min, then increased linearly to 330 °C at a ramping rate of 15 °C·min^−1^. Helium was used as the carrier gas with a constant flow rate of 1 mL·min^−1^ under electronic pressure control.

### Synthesis of TAT

TAT has been a known compound, and its first synthesis was reported by Amemiya *et al*. [26]. TAT was synthesized by the oxidation of 1,2‐pentamethylene azulene with 2,4‐dichloro‐5,6‐dicyanobenzoquinone in high yield. Using the method of Yasunami's azulene synthesis, 1,2‐pentamethylene azulene was prepared from 2H‐cyclohepta[b]furan‐2‐one [30].

## Results

### TAT inhibits proliferation of cancer or immortalized cell lines

**Fig. 1:**
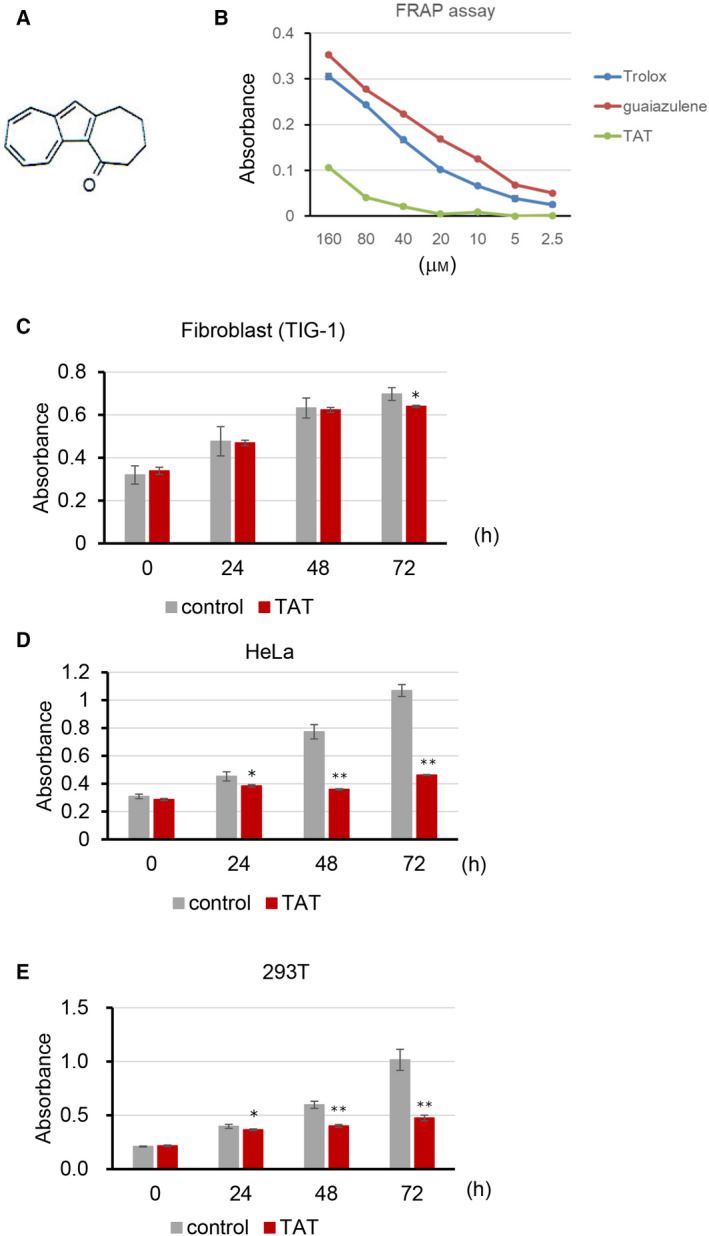
Antioxidant activity and cell viability of TAT‐treated cells. (A) Structure of TAT. (B) FRAP assay to determine the antioxidant capacity of TAT and the positive controls guaiazulene and Trolox. (C–E) WST‐8 assay viability test of normal TIG‐1 fibroblasts, HeLa cancer cells and immortalized 293T cell lines, treated with or without 25 μm TAT. Means ± standard deviation are shown (*n* = 3; *P < 0.05, **P < 0.001, Student's *t*‐test).

TAT is a reddish‐purple compound synthesized from oxidized 1‐alkylazulene originating from guaiazulene (Fig. 1A). Guaiazulene is used as an anti‐inflammatory agent because of its known antioxidant properties; however, the oxidized molecular structure of TAT brings into question its antioxidant properties. In fact, TAT did not exhibit antioxidant activity in a FRAP assay at the level observed for guaiazulene or that of the known antioxidant Trolox (Fig. 1B). To test bioactivity, we administered TAT to three different cultured human cell lines and measured their viability using the WST‐8 assay. TAT inhibited the growth of HeLa cancer cells and the immortalized cell line 293T at concentrations that had no effect on normal TIG‐1 fibroblasts (Fig. 1C–E). These results suggest that TAT has cell proliferation‐inhibitory activity independent of any antioxidant activity.

### TAT induces apoptosis to HeLa cells

**Fig. 2:**
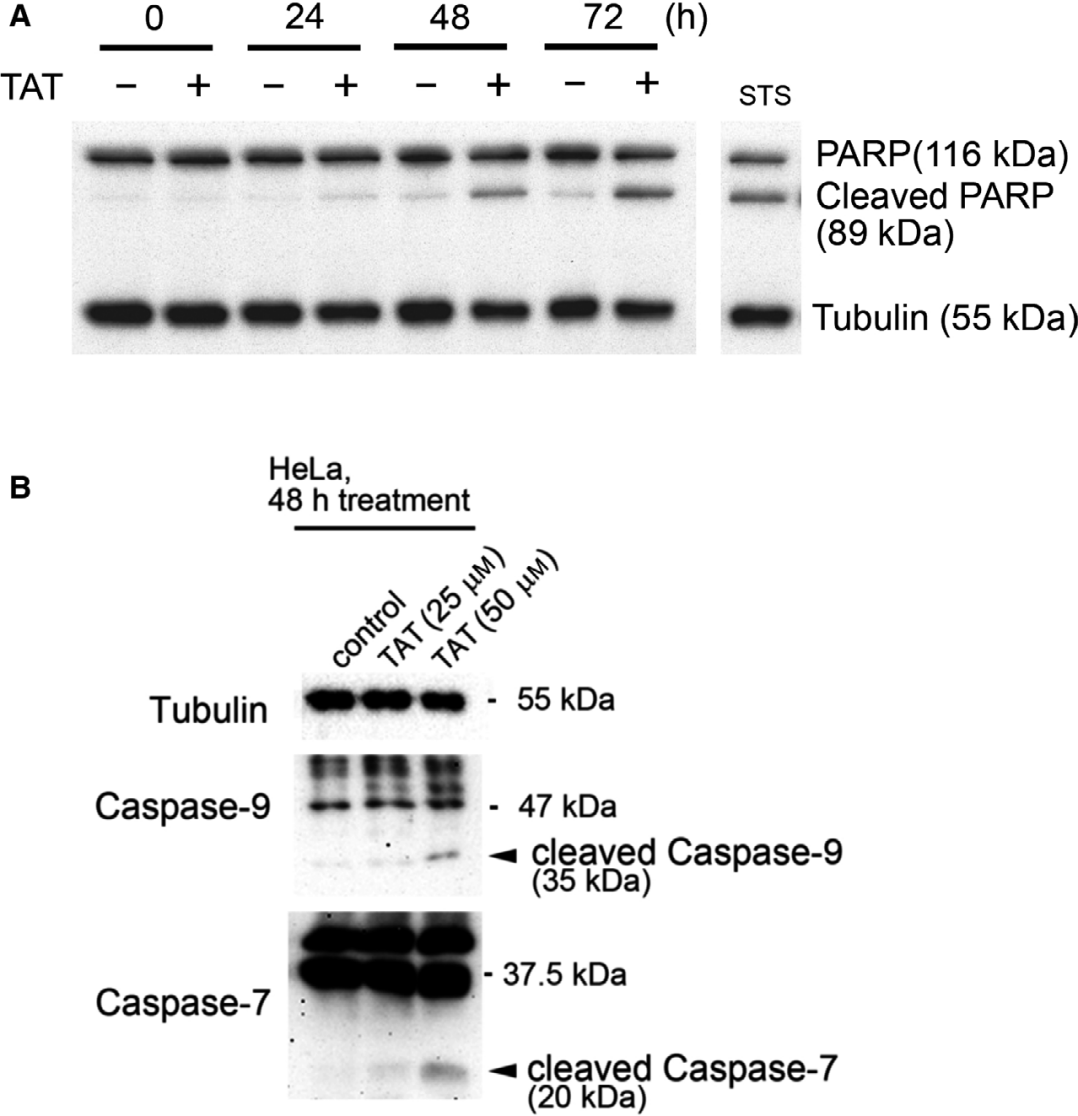
Increased cleavage of PARP and caspases in TAT‐treated cells. (A) Western blot of PARP and its cleavage products in HeLa cells treated with or without 25 μm TAT for each indicated time. Staurosporine was used as the positive control and tubulin as the loading control. (B) Western blots of caspase‐7 and caspase‐9 cleavage products after treatment of HeLa cells with 25 or 50 μm TAT for 48 h.

Whether the inhibition of cell proliferation by TAT was due to cell death was examined by analyzing TAT‐treated HeLa cells every 24 h for cleaved PARP fragments, a marker of apoptosis. PARP cleavage was evident after 48 h in TAT‐treated cells, and it increased further at 72 h (Fig. 2A). In addition, cleavage of caspase‐7 and caspase‐9 was also detected (Fig. 2B). These results suggest that TAT induces cell death in HeLa cells.

### TAT inhibits cellular metabolism

**Fig. 3:**
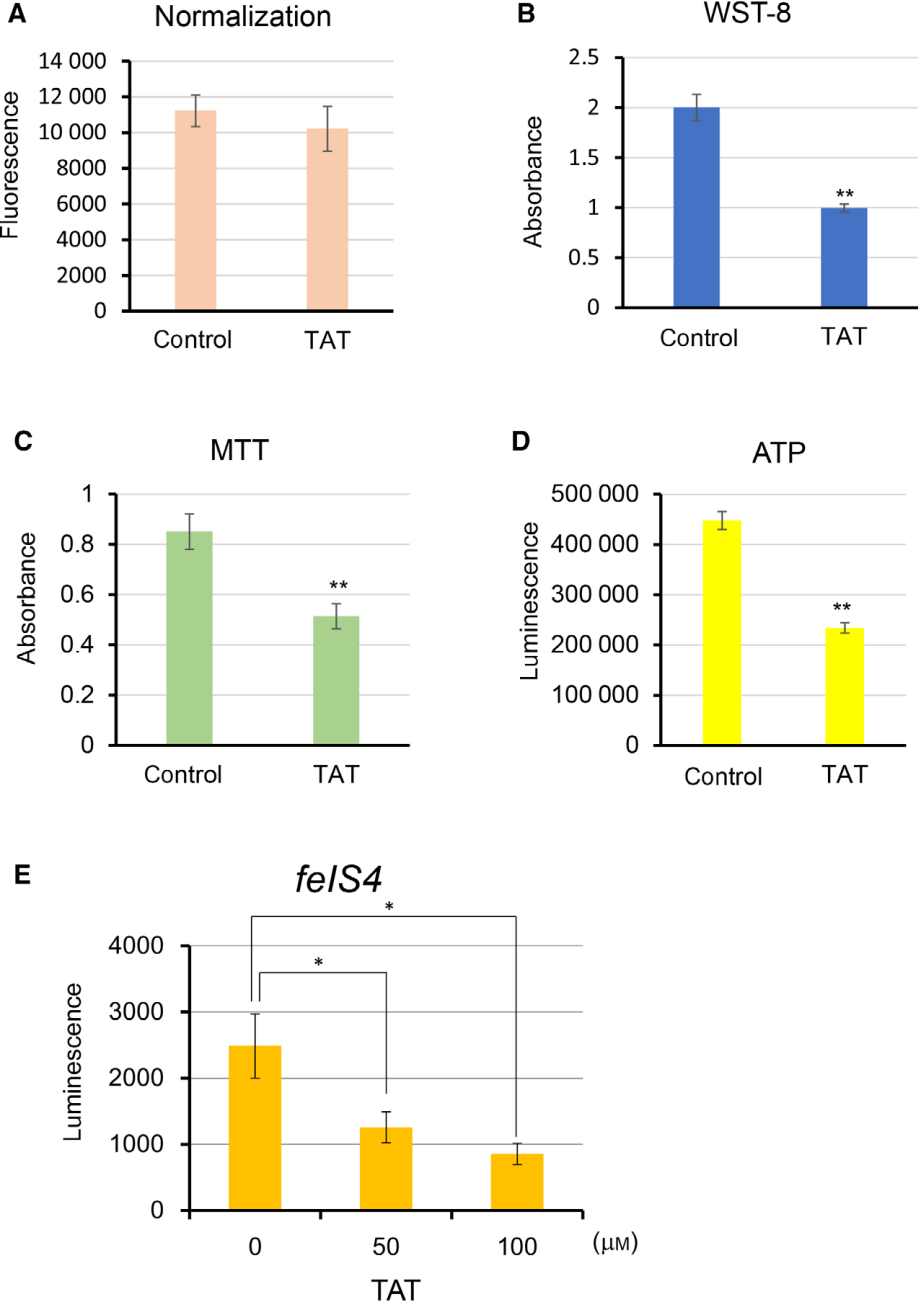
TAT leads to decreased intracellular metabolism and ATP production. (A) Viable cells were measured using a Hoechst 33342 after 48 h at 25 μm TAT‐treated HeLa cells. (B–D) Cell metabolic activity (B, C) and ATP levels (D) of HeLa cells treated with 25 μm TAT were measured after 48 h and simultaneously normalized by the number of viable cells measured by the Hoechst 33342. WST‐8 measures intracellular dehydrogenase activity, and MTT measures mitochondrial reductase activity. (E) Measurement of intracellular ATP as an indicator in the *C. elegans FeIS4* strain, which contains a luciferase transgene, after treatment with varying levels of TAT. Means ± standard deviation are shown (*n* = 3; *P < 0.05, **P < 0.001, Student's *t*‐test).

The effect of TAT on cell death was not as strong as that of known apoptosis‐inducing agents. The WST‐8 cell proliferation test we used was based on intracellular dehydrogenase activity. Generally, this method compares the number of cells with an indicated amount of dehydrogenase activity in each sample, but it also depends on the metabolic status of the cells [31]. Therefore, the number of viable cells was examined using Hoechst 33342, 48 h after the administration of 25 μm TAT. Using this method, we found that TAT treatment did not significantly decrease the number of viable HeLa cells compared with that of control cells (Fig. 3A). When we compared the activity of control and TAT‐treated cells using WST‐8 and MTT assays, which are based on the number of viable cells, we found that in both cases, activity was greatly reduced by TAT (Fig. 3B,C). Intracellular ATP levels were also found to be significantly reduced by TAT compared with that of untreated control cells (Fig. 3D). To confirm that TAT reduces intracellular ATP, we measured the amount of ATP using intracellular ATP as an indicator in the *C. elegans FeIS4* strain, which contains a luciferase transgene [32]. We observed that intracellular ATP decreased in a TAT concentration‐dependent manner (Fig. 3E). In addition, other cancer cell lines, such as MCF7, HepG2 and A549, also decreased the production of WST‐8 and ATP in live cells in a TAT concentration‐dependent manner (Fig. S1). These results suggest that TAT inhibits cellular metabolism before inducing apoptosis.

### TAT influences mitochondrial functions

**Fig. 4:**
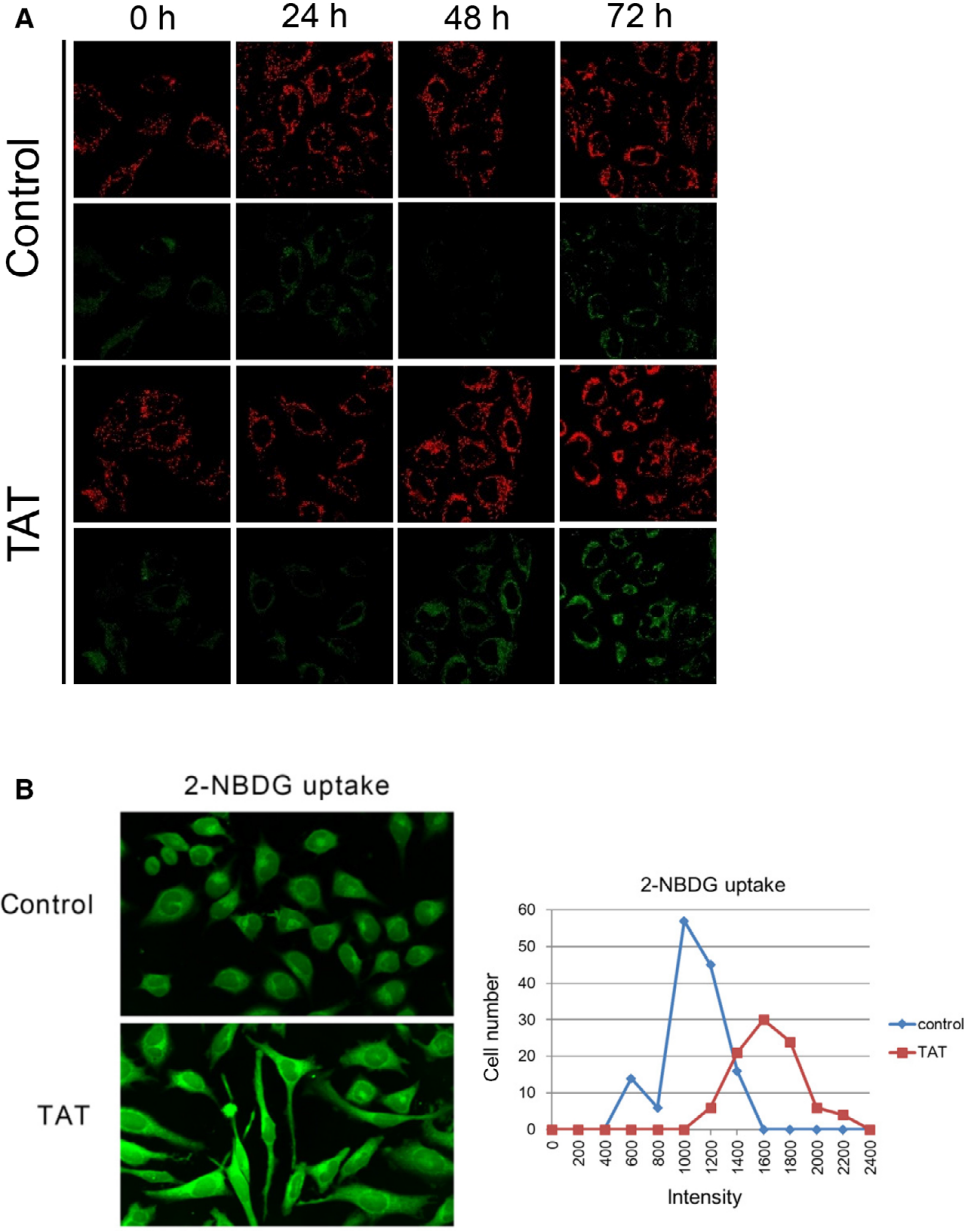
Depolarization of mitochondrial membrane potential by TAT. (A) Mitochondrial membrane potential (ΔψM) was examined in HeLa cells with and without 25 μm TAT treatment at each indicated time using a cationic JC‐1 dye and confocal fluorescence microscopy. Apoptotic or unhealthy cells with a low ΔψM remained in the monomeric form as evidenced by the green fluorescence. (B) After incubation with or without 25 μm TAT for 24 h, the glucose analog 2‐NBDG (400 µm) was introduced for 30 min and uptake examined using confocal fluorescence microscopy. Fluorescence intensity was quantified using ImageJ (developed by Wayne Rasband in NIH, USA).

Because TAT may contribute to the inhibition of cell metabolism, we next investigated the mechanism of action of TAT on metabolism. Caspase‐9 induces apoptosis in a mitochondrial‐dependent manner [33], suggesting that TAT functions in mitochondria. Therefore, we tested the mitochondrial activity of TAT‐treated cells using JC‐1, a mitochondrial membrane potential‐sensitive dye. We observed depolarized mitochondria with time after TAT administration (Fig. 4A), suggesting that TAT impairs mitochondrial function. Impaired mitochondrial function has been reported to enhance glycolysis [12, 34]. To address this, we added 2‐(*N*‐[7‐nitrobenz‐2‐oxa‐1,3‐diazol‐4‐yl]amino)‐2‐deoxyglucose (2‐NBDG), a fluorescent tracer of intracellular glucose analogs, to control and TAT‐treated cells [35] and observed a relative increase in the uptake of 2‐NBDG in the TAT‐treated cells (Fig. 4B). These results suggest that TAT reduces mitochondrial function.

### TAT induces the production of reactive oxygen species in mitochondria

**Fig. 5:**
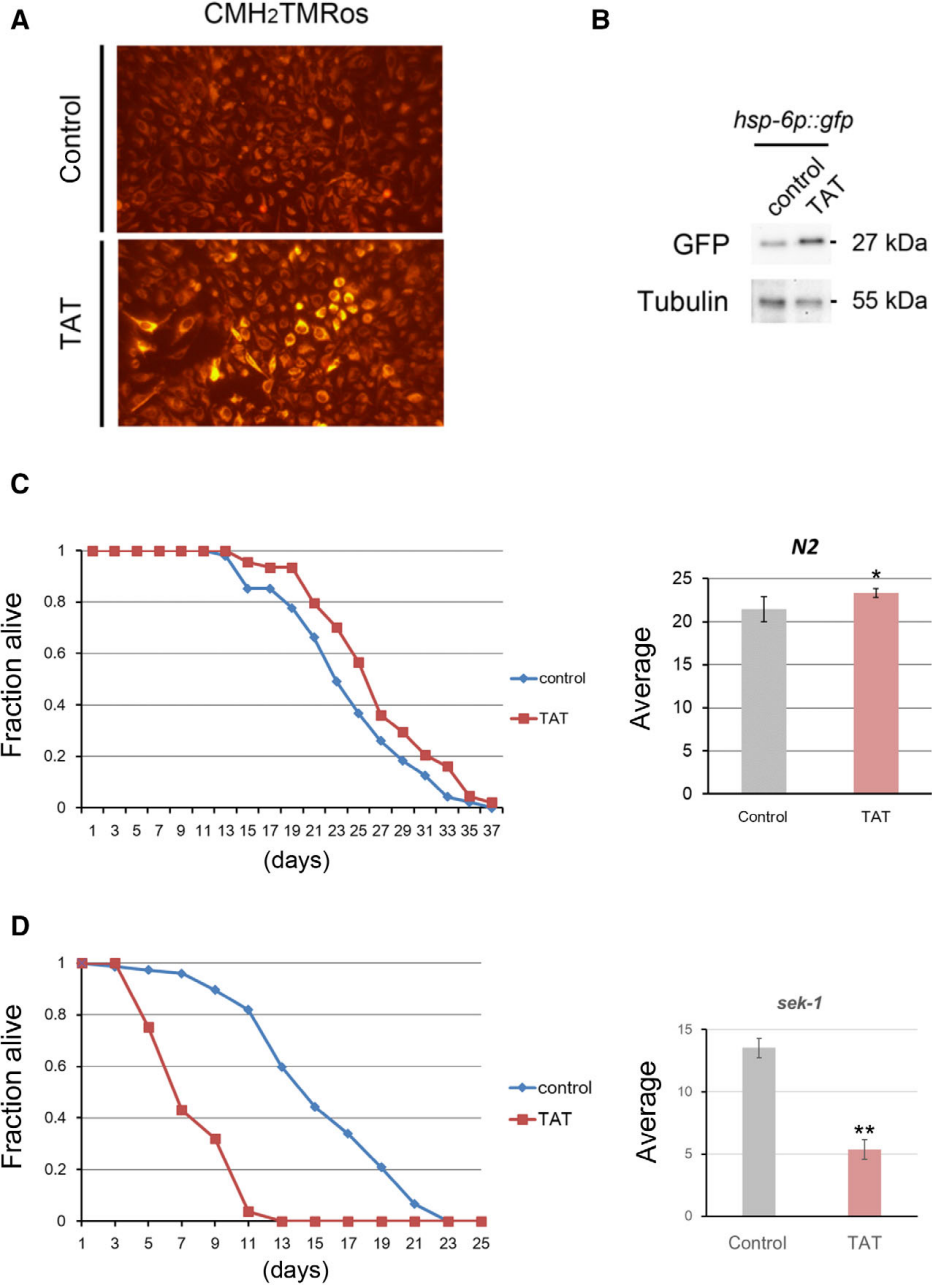
Involvement of TAT in mitochondrial ROS production. (A) HeLa cells treated with or without 25 μm TAT were stained with a reduced form of MitoTracker Orange, CMH_2_TMRos, to detect intracellular ROS using fluorescence microscopy. (B) *C. elegans* carrying the gfp‐tagged *hsp‐6* transgene, which encodes a protein whose expression is up‐regulated in the UPR^mit^, was treated with or without 50 μm TAT for 24 h, and the GFP expression levels were compared by western blotting. Tubulin represents the loading control. (C) 50 μm TAT treatment slightly, but significantly extended the average lifespan of wild‐type N2 *C. elegans* over that of control, untreated worms. (D) 50 μm TAT treatment significantly shortened the average lifespan of oxidative stress‐sensitive *sek‐1* MAPK kinase mutant worms compared with that of controls (*n* = 3; *P < 0.1, **P < 0.05, Student's *t*‐test).

Mitochondrial depolarization reduces electron transfer activity and affects ATP synthesis, and the leak of electrons leads to reactive oxygen species (ROS) production [36, 37]. Therefore, we examined whether TAT acts on mitochondrial activity and generates ROS using the indicator CMH_2_TMRos, which is cleaved by ROS, resulting in fluorescence. We found that fluorescence increased in TAT‐administered HeLa cells compared with that of control cells (Fig. 5A). Mitochondrial abnormalities such as ROS production are known to cause a mitochondrial unfolded protein response (UPR^mit^) [38, 39]. To investigate the involvement of TAT in such a UPR, we administered TAT to transgenic *C. elegans* carrying the gfp‐tagged *hsp‐6* transgene, which encodes a protein whose expression is up‐regulated in the *C. elegans* UPR [40]. Increased expression of GFP was observed in the TAT‐administered worms compared with that of control worms (Fig. 5B). This suggests that TAT causes abnormalities in mitochondria, leading to the development of ROS and subsequent induction of unfolded proteins. Moderate mitochondrial ROS development has been reported to extend the lifespan of *C. elegans* [41]. The lifespan of TAT‐administered wild‐type N2 worms was slightly extended (Fig. 5C). The p38 mitogen‐activated protein kinase (MAPK) pathway is involved in the elimination of intracellular ROS [42]. TAT treatment significantly reduced the lifespan of the *C. elegans sek‐1* MAPK kinase mutant, which is sensitive to oxidative stress (Fig. 5D). This finding supports the involvement of TAT in the production of mitochondrial ROS.

### TAT inhibits mitochondrial electron transfer complex II

**Fig. 6:**
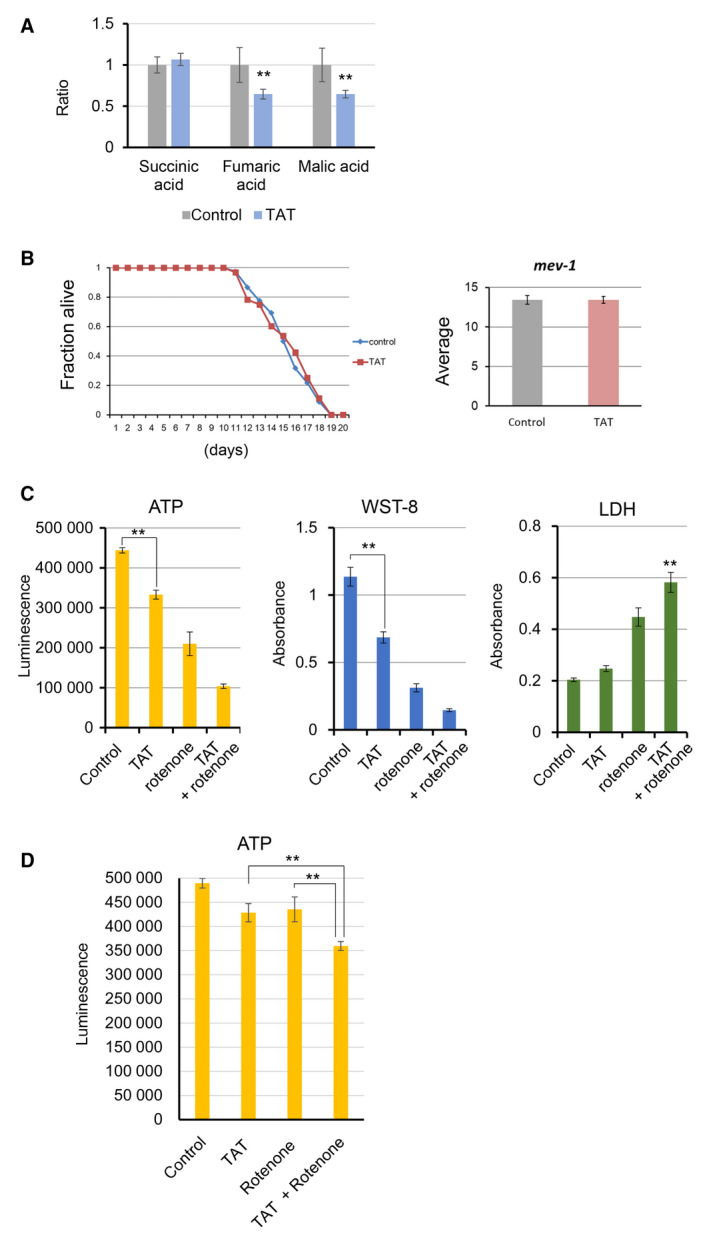
TAT acts on electron transfer complex II. (A) TAT treatment reduced fumaric and malic acid levels in HeLa cells, while succinic acid was unchanged relative to that of untreated controls (*n* = 4; **P < 0.05, Student's *t*‐test). (B) The lifespan of the typically short‐lived and oxygen‐hypersensitive *C. elegans* mutant, *mev‐1*, which encodes the SDH subunit of electron transfer complex II, was measured after 50 μm TAT treatment and the average compared with that of control, untreated *mev‐1* worms (*n* = 3; *P* = 0.99, Student's *t*‐test). (C) TAT cooperates with the electron transfer complex I inhibitor, rotenone, to inhibit cellular energy metabolism. HeLa cells were treated with or without TAT (25 μm) and rotenone (0.25 μm) for 48 h, and then ATP levels, WST‐8 and lactate dehydrogenase were measured, respectively. (D) TAT inhibits ATP synthesis in isolated mitochondria in cooperation with rotenone. Isolated mitochondria were treated with or without TAT (25 μm) and rotenone (0.5 μm) at 37°C for 10 min in reaction buffer. Then ATP levels were measured by adding ATP luminescence reagent.

Next, we examined the specific site of TAT inhibition within the mitochondria. To investigate this, we measured the concentration of each metabolite in the central metabolic pathway by GC/MS after trimethylsilylating the extracts of TAT‐administered cells. The concentration of succinate did not change significantly after TAT treatment, whereas fumarate and malate were significantly reduced relative to control values (Fig. 6A). Furthermore, to study the effects of TAT *in vivo*, we used the short‐lived and oxygen‐hypersensitive *C. elegans* mutant, *mev‐1*, which encodes the succinate dehydrogenase (SDH) subunit of electron transfer complex II. The lifespan of the *mev‐1* worms was not altered by TAT as compared with that of the untreated *mev‐1* worms (Fig. 6B). Subsequently, we investigated the inhibitory effect of TAT on energy metabolism in cultured cells using rotenone, an electron transport chain complex I inhibitor. TAT and rotenone each reduced the amount of intracellular ATP. Furthermore, the simultaneous administration of TAT and rotenone significantly reduced the amount of intracellular ATP. Together, the value of lactate dehydrogenase, which is an index of cytotoxicity, increased significantly (Fig. 6C). In addition, we performed an *in vitro* assay using isolated mitochondria to determine whether the inhibition of ATP synthesis occurred by TAT. The amount of ATP synthesis in isolated mitochondria was inhibited by TAT and rotenone, respectively. ATP synthesis was more strongly inhibited by simultaneous administration of TAT and rotenone (Fig. 6D). These results suggest that TAT inhibits the mitochondrial electron transfer complex II.

## Discussion

Reports on the bioactivity of guaiazulene and its derivatives are lacking with respect to their mechanisms of action. In this study, we found that TAT, a guaiazulene derivative, inhibited oxidative phosphorylation by blocking mitochondrial electron transfer complex II. Therefore, we have revealed for the first time the bioactivity of guaiazulene derivatives, other than their antioxidant activity, by analyzing cancer cell lines and a model animal, *C. elegans*.

It is generally accepted that the Warburg effect is responsible for ATP production via glycolysis, rather than mitochondrial oxidative phosphorylation, in cancer cells even under aerobic conditions. However, oxidative phosphorylation also occurs and can sufficiently provide ATP in cancer cells under aerobic conditions. Thus, an anticancer strategy that acts on mitochondria to inhibit oxidative phosphorylation and deplete the energy of cancer cells could be effective against cancer cells with a high‐energy metabolism [8]. Our results show that TAT‐treated cells initially showed a significant decrease in metabolism and eventually induced apoptosis. TAT caused mitochondrial depolarization in cells and produced ROS. Furthermore, we found that GC/MS reduced fumaric acid and malate in cells during TAT treatment. Because succinate is oxidized to fumarate by SDH in electron transport complex II, the decrease in fumarate is thought to be caused by a decrease in the function of electron transport complex II. The fact that the lifespan of *mev‐1* worms, which encodes SDH, was not altered by TAT supports that TAT acts on electron transfer complex II. Furthermore, TAT synergistically inhibited ATP production with rotenone, an inhibitor of electron transfer complex I. This result also supports the idea that TAT acts on electron transfer complex II. The production of ROS in mitochondria causes mitochondrial depolarization and inhibits oxidative phosphorylation [43]. Therefore, TAT may induce mitochondrial depolarization by inhibiting mitochondrial electron transfer complex II and promoting ROS production. Because moderate mitochondrial production of ROS has been reported to extend the lifespan of *C. elegans* [41], it is plausible that TAT causes an extension of the lifespan in wild‐type N2 worms by the same mechanism.

TAT induces apoptosis in cancer cells and immortalized cell lines, but similar concentrations of TAT do not induce cell death in normal cells, presumably because their ATP requirement is lower than that of cancer cells [44]. Therefore, in cancer cells with high ATP consumption, inhibition of mitochondrial oxidative phosphorylation by TAT may deplete intracellular energy and cause cell death. TAT is relatively easy to synthesize, consisting of only C, H, and O, and the production of more potent analogs with anticancer activity is expected. Furthermore, TAT is projected to be tested as a low‐cost compound with anticancer activity in mouse tumor models and in combination with other anticancer agents.

## Conflict of interest

The authors declare no conflict of interest.

## Author contributions

CK and HI conceived and planned the experiments. JY synthesized TAT and helped supervise the project. HI wrote the manuscript with support from CK. JY and HI conceived the original idea.

## Supporting information


**Fig. S1**. TAT decreases metabolism in several cancer cell lines.

## Data Availability

All data generated or analyzed during this study are included in this published article.
