# Supplementary material for: Guaiazulene derivative 1,2,3,4‐tetrahydroazuleno[1,2‐b] tropone reduces the production of ATP by inhibiting electron transfer complex II
Source: FEBS Open Bio. 2021 Sep 21;11(11):2921–32. doi: 10.1002/2211-5463.13215 (PMC8564332; doi:10.1002/2211-5463.13215)
Supplement: Supplementary file 1 — Fig. S1. TAT decreases metabolism in several cancer cell lines. [file FEB4-11-2921-s001.pdf]

Sup. Fig. 1

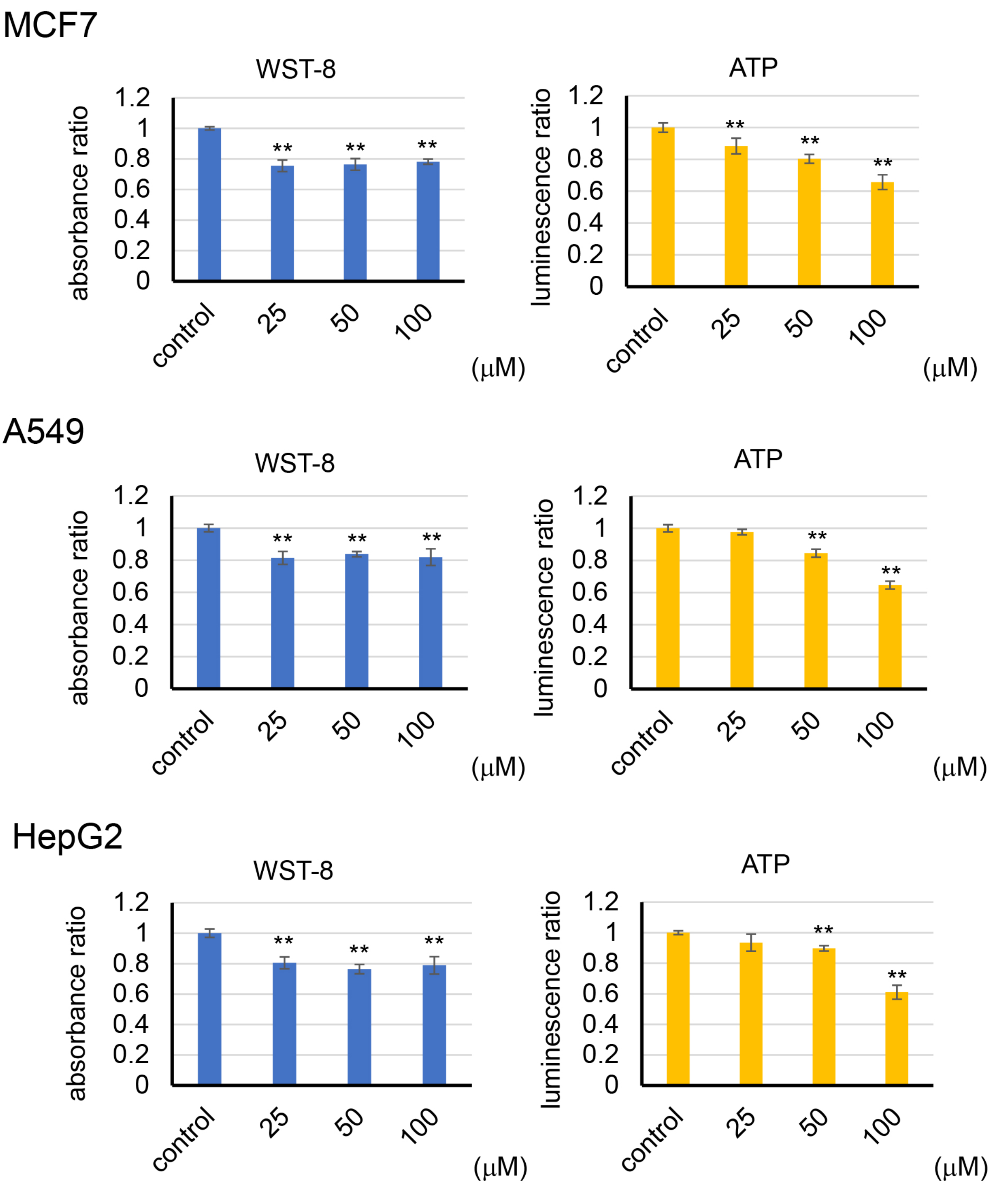

Supplementary Fig. 1 TAT decreases metabolism in several cancer cell lines

Measurement of cellular metabolism after 48 hours of the culture of cancer cell lines treated with 25, 50, or 100 M TAT. The ratio of viable cells to total cell number using a Normalization kit and Hoechst 33342. The absorbance (WST-8) and luminescence (ATP) were normalized with viable cells measured by the normalization kit and the ratio to the control was calculated. Means ± SD are shown (n = 3; **P < 0.001 Student's t-test)
